# Seeking out the primary tumour: multi-modality imaging of metastatic cardiac angiosarcoma

**DOI:** 10.1093/ehjimp/qyaf130

**Published:** 2025-10-24

**Authors:** Ryan Karlsson, Michael Cronin, Cian Murray, Roger Byrne

**Affiliations:** Department of Cardiology, St James’s Hospital, James St, Dublin D08 NHY1, Ireland; Department of Cardiology, Mater Private Network, Dublin, Ireland; Department of Cardiology, University Hospital Waterford, Waterford, Ireland; Department of Cardiology, Mater Private Network, Dublin, Ireland

**Keywords:** cardiac angiosarcoma, multimodality imaging, magnetic resonance imaging, intracardiac echocardiography, cardiac biopsy

## Case

**Figure 1:**
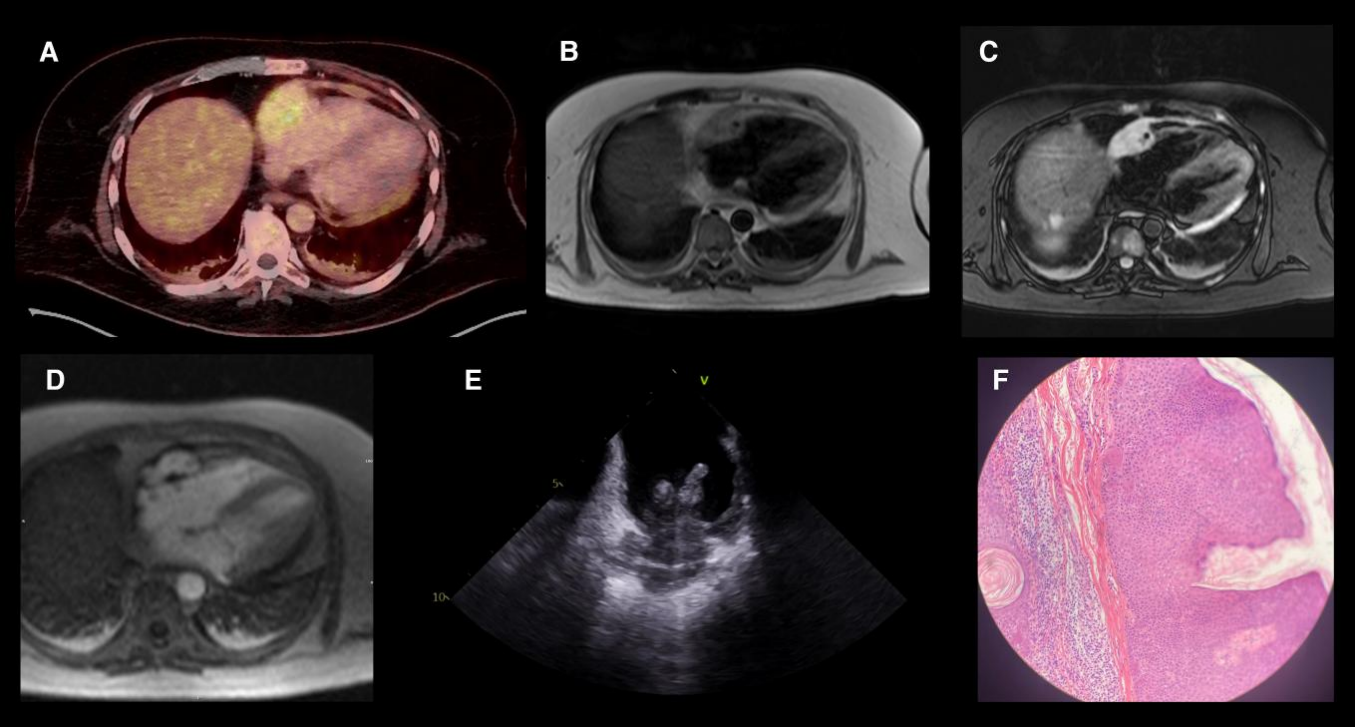
Multi-modality imaging of primary cardiac angiosarcoma. (*A*) Axial FDG-PET image displaying avid uptake in the right atrium. (*B*) Axial T2-weighted CMR image showing an isointense mass within the right atrium. (*C*) Axial T2-weighted fat-suppressed CMR image demonstrating absence of fat signal within the lesion. (*D*) Axial first-pass perfusion CMR image showing diffuse, intense enhancement of the mass. (*E*) Intra-cardiac echocardiography image depicting the right atrial mass, providing guidance for tissue biopsy. (*F*) Histopathology of the right atrial biopsy showing atypical spindle cells within a collagenous stroma.

A 44-year-old male with hypertension presented with exertional dyspnoea. Blood tests demonstrated anaemia (haemoglobin 12.3 g/dL; reference 13.0-18.0 g/dL) and elevated D-dimer (7.4 u/mL; reference <0.5 u/mL). CT pulmonary angiography revealed extensive lytic bone lesions and a large pericardial effusion, which was further assessed with transthoracic echocardiography (*see*  Supplementary data online, *Video S1*). Pericardiocentesis was non-diagnostic. Whole-body magnetic resonance (MR) imaging revealed hepatic and splenic metastases. Following two inconclusive metastatic biopsies, positron-emission tomography was performed revealing additional FDG-avid soft tissue lesions in the right atrium (*Figure 1A*) and left thigh. Cardiac MR further characterized a 25 × 50 × 35 mm mass with central necrosis located along the right atrial free wall extending into the appendage and atrioventricular groove with associated pericardial thickening and right coronary encasement (*see*  Supplementary data online, *Video S2-S4*). The lesion showed T2-weighted isointensity (*Figure 1B*), lack of fat saturation (*Figure 1C*) and diffuse enhancement on first-pass perfusion (*Figure 1D*; Supplementary data online, *Video S5*) along with heterogeneous late gadolinium uptake (*see*  Supplementary data online, *Video S6-S7*) raising the differential of cardiac angiosarcoma, lymphoma or paraganglioma. Following four further non-diagnostic extra-cardiac biopsies, intra-cardiac echocardiography-guided sampling of the right atrial mass was performed (*Figure 1E*; Supplementary data online, *Video S8*). Microscopy revealed atypical spindle cells within a collagenous stroma (*Figure 1F*) and immunohistochemistry was positive for vimentin and CD34, and negative for smooth muscle actin, desmin, cytokeratins and S100, confirming the diagnosis of metastatic right atrial angiosarcoma. The patient was referred for oncological assessment but declined palliative chemotherapy owing to the anticipated side-effect profile, electing to transition to hospice care.

Cardiac angiosarcoma, although exceptionally rare, represents the most common primary malignant cardiac tumour. It typically arises from the right atrial free wall as an infiltrative, lobulated, heterogeneous mass often with associated haemorrhagic pericardial effusion. Characteristic CMR features include heterogeneous T1 and T2 signals, intense but heterogeneous early enhancement on first-pass perfusion, and patchy late enhancement, with necrotic and haemorrhagic areas. PET-CT typically demonstrates intense, heterogeneous FDG uptake with frequent metastatic disease. In comparison, cardiac lymphoma enhances mildly and homogeneously and tends to encase rather than invade, while paraganglioma shows avid, homogeneous early enhancement, is well-circumscribed, and most often involves the left atrium.

While CMR tissue characterization can reliably distinguish angiosarcoma from benign mimics such as myxoma histological confirmation with biopsy remains essential, as lymphoma or metastasis from a distant site may display similar imaging features but differ fundamentally in management. Early diagnosis is critical, as prognosis is poor due to rapid local invasion and early metastasis. Timely detection may allow complete surgical resection, which confers greater survival. Biopsies of metastatic angiosarcoma lesions may unfortunately appear deceptively bland or be non-diagnostic due to necrosis. Intra-cardiac echocardiography provides valuable guidance when primary tumour sampling is required.

## Supplementary Material

qyaf130_Supplementary_Data

## Supplementary data

**Funding:** There was no funding involved in this case report. This study did not receive any specific grant from any funding agency in the public, commercial, or not-for-profit sectors.

**Consent:** The authors confirm that written informed consent for submission and publication of this case report including images and associated text has been obtained from the patient in line with the Committee on Publication Ethics (COPE) guidance.

**Data availability:** No new data were generated or analysed in support of this research.

## Lead author biography

Dr Ryan Karlsson, MB BCh BAO MRCPI, graduated from Trinity College Dublin in Ireland. He is currently working as a Cardiology Registrar at St James's Hospital Dublin. He has a broad range of interests within cardiology including cardiac imaging and heart failure.

